# Acute appendicitis and the role of pre-operative imaging: A cohort study

**DOI:** 10.1016/j.amsu.2020.10.008

**Published:** 2020-10-09

**Authors:** Jaideep Singh Rait, Jirayr Ajzajian, Joshua McGillicuddy, Amit Sharma, Brian Andrews

**Affiliations:** a William Harvey Hospital, East Kent NHS Trust, Kennington Rd, Willesborough, Ashford, TN24 0LZ, United Kingdom; b Darent Valley Hospital, Dartford and Gravesham NHS Trust, Dartford, DA2 8DA, United Kingdom; c Medway Maritime Hospital, Medway NHS Trust, Windmill Road, Gillingham, Kent, ME7 5NY, United Kingdom

**Keywords:** Appendicectomy, Pre-operative imaging, Negative appendicectomy, Ultrasonography, Computed tomography

## Abstract

**Introduction:**

Acute right iliac fossa (RIF) pain is amongst the most common presentation to the surgical team. Acute appendicitis is the most common cause of this pain and often warrants surgical intervention. In many cases intervention results in a negative appendicectomy with unnecessary complications as a result. The aim of this study was to compare the efficacy of pre-operative imaging in the diagnosis of acute appendicitis to reduce the rate of negative appendicectomy and unnecessary operative intervention.

**Methods:**

A retrospective single centre cohort study was undertaken in a district general hospital (DGH) of all laparoscopic appendicectomies over a six-year period. 1344 cases were included and were examined for the use of pre-operative imaging (and type) or none. The sensitivity, specificity, negative and positive predictive value for each type of imaging were analysed for their accuracy in diagnosis appendicitis based on the final histological analysis.

**Results:**

The negative appendicectomy rate was found to be greatest in those undergoing ultrasonography (48.21%) as their method of pre-operative imaging whilst those who underwent computed tomography (CTAP 20.26%) had a lower rate equivalent to that of clinical diagnosis alone (20.73%).

**Conclusion:**

USS is less sensitive than CT in diagnosing acute appendicitis. There is no statistically significant difference in negative appendicectomy rate between clinical diagnosis and CT diagnosis. Pre-operative imaging has a role in the diagnosis of appendicitis but needs to be utilised appropriately to reduce the strain on the surgical department and prevent the potential of a negative appendicectomy.

## Highlights

• Pre-operative imaging has a role in the diagnosis of acute appendicitis
• The selection of the appropriate modality of imaging should be carefully selected to maintain a low negative appendicectomy rate
• Clinical diagnosis alone may be sufficient in certain cases of acute appendicitis

## 1 Introduction

Acute onset of pain in the right iliac fossa (RIF) is a common presentation that is frequently referred to the general surgical team in the secondary care setting [1]. Amongst the causes of RIF pain, acute appendicitis is the most common indication for emergency surgical intervention, in the USA and UK there are 250,000 and 35,000 cases reported annually. Making a diagnosis of acute appendicitis has frequently been considered to be a clinical endeavour [2], however in an effort to reduce the negative appendicectomy rate (NA) [3] clinicians are aided by scoring systems [4] and imaging such as ultrasound [5], computerised tomography and MRI [6].

Although USS is non-invasive, it is highly operator dependent and has a lower sensitivity than CT [7]. Whilst CT has a greater sensitivity but does require significant exposure to radiation (approximately equivalent to 200 chest radiographs [8]) and the use of nephrotoxic intravenous contrast agents which are potentially harmful.

According to the Right Iliac Fossa Pain Treatment (RIFT) study, in the UK the rate of negative appendicectomy in women aged 16–45 was 28.2%. The rate is lower in men but nevertheless significant. The percentage of negative appendicectomy for men in the same age group is 12.1%. These rates are substantially higher than other high-income countries [9].

The primary aim of this study was to compare the sensitivity, specificity, positive predictive value and negative predictive values of USS and CT in making a diagnosis of acute appendicitis. The secondary aim s were to compare the role of pre-operative imaging in reducing NA rate (and therefore unnecessary invasive surgical intervention) against a cohort of patients who underwent surgical intervention on purely clinical grounds.

## 2 Methods

A retrospective single centre cohort study was undertaken in a UK district general hospital which provides paediatric (for those aged over five years) and adult general surgical services. The study population was established from all patients who underwent a laparoscopic procedure over a six-year period (2012–2018) this population was then refined to include only those who underwent a laparoscopic appendicectomy as coded in the electronic theatre database. The results of pre-operative imaging (on the index admission) and final histology reports for each case were analysed on a post hoc basis and compared.

In this institution, pre-operative imaging was limited to ultrasonography (USS) and computerised tomography (CT) either CT abdomen and pelvis (CTAP) or CT kidneys ureter and bladder (CTKUB).All ultrasound examinations were performed by suitably trained radiographers, all CT scans were reported by consultant radiologists, and all appendix specimens were reported by a consultant histopathologist. To allow for comparisons between the different imaging modalities, the results of pre-operative imaging were grouped into: those that clearly stated appendicitis (whether complicated or uncomplicated), those that were completely normal and those that demonstrated an alternative pathology or were inconclusive. Histological samples were grouped into: those with signs of acute appendicitis (either inflamed or gangrenous), those that were entirely normal and those samples which included an alternative pathology (e.g. neuroendocrine tumour or faecolith). Ethical approval was not sought as all data collected was anonymised and research was registered (UIN: researchregistry6001). All work has been reported in line with the STROCSS criteria [10].

### 2.1 Statistical analysis

Data was analysed through the use of Microsoft excel and statistical tests were employed through the use of MedCalc software. Diagnostic test evaluation was performed to obtain results for sensitivity, specificity, negative predictive value and positive predictive value (see Table 6). The data was then analysed via an unpaired *t*-test to determine the ability to reject the null hypothesis (that pre-operative imaging reduces the NA rate).

## 3 Results

**Fig. 1:**
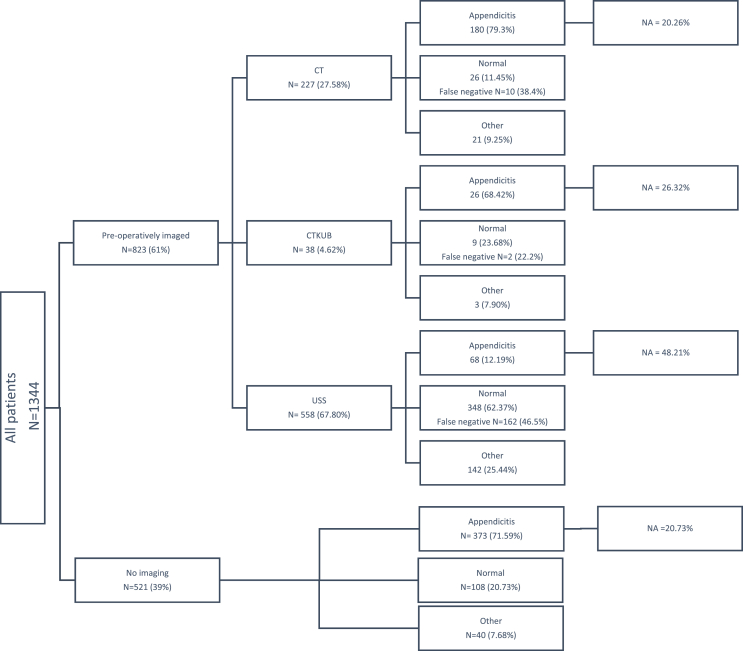
Flow diagram summarising the radiological investigations and respective histological findings for the patient groups. (N = number of patients in each arm).

A flow diagram detailing the diagnostic pathway of the 1344 patients included in the study, and their histological diagnoses is presented in Fig. 1, demographic data of these patients is presented in Table 1.

**Table 1:** Demographic data of the patients who underwent laparoscopic appendicectomy between 2012 and 2018.

Gender	Laparoscopic appendicectomies	Median age (years)
Female	893 (66.4%)	30
Male	451 (33.6%)	32

### 3.1 Appendicitis diagnosed without pre-operative imaging

A total of 521 patients underwent laparoscopic appendicectomy based solely on a clinical evaluation, of which 373 (71.59%) patients, (219 males and 154 females), had acute appendicitis confirmed on final histological examination. The clinical diagnosis alone correctly diagnosed acute appendicitis in 78.49% of males and only 63.64% of females.

In 40 (7.68%) patients the biopsy resulted in the diagnosis of an alternative pathology; carcinoid tumour or the presence of faecolith (not causing inflammation).

However, histological examination of the appendix was normal (Negative appendicectomy) in 108 (20.73%) patients (15.41% of males and 26.85% of females). These results are tabulated in Table 2.

**Table 2:** Histological results of the patients who underwent laparoscopic appendicectomy without pre-operative imaging according to gender.

Histological result	Male	Female	Total
**Normal**	43 (15.41%)	65 (26.85%)	108 (20.73%)
**Appendicitis**	219 (78.49%)	154 (63.64%)	373 (71.59%)
**Other**	17 (6.09%)	23 (9.50%)	40 (7.68%)
**Total**	279	242	521

### 3.2 Patients who underwent pre-operative imaging

#### 3.2.1 CT abdomen and pelvis

227 patients (106 male, 121 female) underwent a pre-operative diagnostic CT scan of the abdomen and pelvis (CTAP), yielding results of acute appendicitis in 180, a normal appendix in 26, and alternative pathology in 21.

Of the 180 patients with a CTAP diagnosis of acute appendicitis, only 159 (88.33%) had this diagnosis confirmed histologically. Of the 26 patients whose appendix appeared normal by radiological criteria, 10 (38.4%) were subsequently diagnosed with acute appendicitis on histological examination. Of the 21 who had “other pathology in the appendix” as reported on CTAP according to CTAP, 9 (42.86%) had normal appendices, and 12 (57.14%) were subsequently found to have acute appendicitis on histological examination.

The negative appendicectomy in the group of patients who underwent a pre-operative CTAP was 20.26%.

These results of patients who had a pre-operative CTAP are tabulated in Table 3.

**Table 3:** Correlation of radiological and histological diagnoses in the CTAP group.

CTAP Result	Normal		Appendicitis		Other		Total	Histological result/imaging result (%)
N	26		180		21		227	
Histology result: Normal	16	61.54%	21	11.67%	9	42.86%	46	20.26%
Histology result: Appendicitis	10	38.46%	159	88.33%	12	57.14%	181	79.74%

#### 3.2.2 CTKUB

38 patients (23 female, 15 male) underwent a pre-operative CT kidney-ureter-bladder (CTKUB), because right sided renal colic was initially suspected at presentation. A radiological diagnosis of acute appendicitis was made in 26 patients of which 25 (96.15%) had the diagnosis confirmed histologically. Of the nine patients whose appendix was reported as “Normal” on CTKUB, 2 (22.22%) had histologically proven appendicitis.

The negative appendicectomy rate in the CTKUB group was 26.32%.

The results of the CTKUB group are tabulated in Table 4.

**Table 4:** Correlation of radiological and histological diagnoses in the CTKUB group.

CT CTKUB Result	Normal		Appendicitis		Other		Total
N	9		26		3		38
Histology result: Normal	7	77.78%	1	3.85%	2	66.67%	10
Histology result: Appendicitis	2	22.22%	25	96.15%	1	33.33%	28

#### 3.2.3 USS

A total of 558 patients (51 male, 507 female) underwent USS prior to undergoing a laparoscopic appendicectomy. This group had a negative appendicectomy rate of 48.21%. The results are shown in Table 5.

**Table 5:** Radiological and histological results of the patients who underwent USS before laparoscopic appendicectomy.

USS Result	Normal		Appendicitis		Other		Total
N	348		68		142		558
Histology result: Normal	186	53.45%	14	20.59%	69	48.59%	269
Histology result: Appendicitis	162	46.55%	54	49.41%	73	51.41%	289

**Table 6:** Sensitivity, specificity, positive predictive value and negative predictive value of CTKUB, CTAP and USS in detecting appendicitis.

Type of imaging	Sensitivity	95% Confidence Interval	Specificity	95% Confidence Interval	Positive predictive value %	95% Confidence Interval	Negative predictive value %	95% Confidence Interval
CTKUB	**90.32**	74.25–97.96	**90.00**	55.50–99.75	**96.55**	81.29–99.45	**75.00**	50.09–89.97
CTAP	**89.16**	84.05–93.08	**54.35**	39.01–69.10	**89.60**	86.24–92.22	**53.19**	41.40–64.64
USS	**55.15**	50.78–59.47	**94.80**	91.42–97.13	**95.38**	92.49–97.19	**52.04**	49.57–54.50

The NA rate and statistical significance between those that were pre-operatively imaged and those who underwent the various forms of imaging are shown in Table 7.

**Table 7:** NA rate for no pre-operative imaging, CT-CTKUB, CTAP and USS for suspected appendicitis patients.

Modality	NA rate (%)	Significance level (P value)
No pre-operative imaging	20.73	–
CTKUB	26.32	0.4170
CT AP	20.26	0.8839
USS	48.21	<0.0001

## 4 Discussion

Appendicitis is the most common presentation of acute abdomen to the secondary care setting in the UK and approximately 10% of the population will develop acute appendicitis in their lifetime [11]. In addition to this, time delay to intervention may increase the risk of rupture [12] and therefore timely diagnosis and intervention is vital to prevent complications for this common presentation.

We analysed the sensitivity, specificity, positive predictive value, negative predictive value and NA rate amongst those who were pre-operatively imaged and those who were not.

Amongst those who were not pre-operatively imaged and taken to theatre for laparoscopic appendicectomy based on clinical suspicion alone consisted of 521 patients with 373 (71.5%) with confirmed appendicitis on histopathological analysis. This demonstrates a NA rate of 20.73%. This NA rate is in line with other previously published studies [13,14].

Those who were pre-operatively imaged were separated into the imaging modality undertaken (CTKUB/CT AP/USS). CT AP consisted of 227 patients with 181 (79.74%) having evidence of appendicitis on histological analysis and a NA rate of 20.26%. The CTKUB group consisted of 38 patients over the study period with 28 (76.38%) of these having appendicitis as their final histological diagnosis and a NA rate of 26.3%. Finally, USS was the largest group with 558 patients and 289 (51.79%) showing histological evidence of appendicitis and an NA rate of 48.2% (Table 8).

**Table 8:** Summary of the diagnostic approaches and respective negative appendicectomy rates.

Diagnostic approach	Number of patients (N)	Histologically proven appendicitis (N)	Negative Appendicectomy (%)
Clinical diagnosis	521	373	20.73%
CTAP	227	181	20.26%
CTKUB	38	28	26.3%
USS	558	289	48.2%

The results of the study reflect those borne out of clinical experience in that USS is less sensitive than CT in diagnosing appendicitis. We found that USS had a sensitivity of 55.15% (CI, 50.78–59.47) and specificity of 94.8% (CI, 91.42–97.13) which indicated a worse sensitivity but better specificity than other previously published studies [15]. We believe our data to be more in line with actual findings as achieving a higher sensitivity level is difficult and highly operator dependent [7]. Overall, this low sensitivity value is likely the cause behind the high NA rate (48.2%, p-<0.0001); such that many that undergo USS which fails to show positive or negative findings will likely require a diagnostic laparoscopy in which most surgeons continue to laparoscopic appendicectomy due to a lack of guidance on the management of a normal macroscopic appendix [16]. Additionally, the gender split in those who were not pre-operatively imaged or underwent CT was equal whereas those that underwent USS demonstrated were in the majority female. This highlights the gender inequality of USS and demonstrates that as this group had a high NA rate that more female patients had an unnecessary operation.

We found that NA rate in patients with perioperative CT was not statistically different from the patients who had no perioperative imaging (Table 7). We were able to show that CTKUB in our study had a high level of sensitivity (90.32 CI, 74.25–97.96) and specificity (90 CI, 55.50–99.75). Our study also showed that CTKUB had a high positive and negative predictive value, thus reflecting the reliability of the test. CT AP in contrast had an equally high sensitivity (89.16 CI, 84.05–93.08) but a lower specificity (54.35 CI, 39.01–69.10) thus showing that CT AP was a reliable test for correctly diagnosing appendicitis but not as useful as excluding those without the condition. This is also reflected in the high positive predictive value (89.6 CI, 86.24–92.22) and low negative predictive value (53.19 CI, 41.40–64.64). Our study was not in line with published data [15,17] for specificity for CT and this may be because we separated patients undergoing CT into CTKUB and CTAP.

Interestingly, a number of patients (29) initially underwent USS followed by CT. This subgroup was found to have an NA rate of 34.5%, which is intermediate between USS (48.20%) and CT (20.26%) and therefore shows CT may alleviate the high NA rate of USS. However, due to low numbers of such patients, such findings cannot be verified.

There are certain limitations of this study. The retrospective design involves selection bias and a prospective design would allow all those presenting with RIF pain to be included. This is compounded by the fact that the data included represents only those who underwent a laparoscopic procedure and excluded those who may have had an open operation. This may represent a further element of selection bias but as currently the majority of patients undergo laparoscopic procedures over open operations this would likely have been minimal.

The authors propose that USS is reserved for those patients for whom appendicitis has been ruled out and to look for alternative pathology. The optimum investigation as shown by this study would be one based on no pre-operative imaging or CTKUB as a method to provide the minimum amount of radiation and no nephrotoxic contrast agents to visualise the appendix. This method would reduce the NA rate as it would rely on clinical judgement which in this study has been shown to have an acceptable NA rate in line with the national average or on a low dose, no contrast method of CT.

Nevertheless, while making a decision about the diagnostic approach for possible appendicitis, the risks and benefits of the diagnostic tools should be assessed. Radiological investigations come with risk of radiation and in certain population groups such as pregnant women and young patients it is strictly discouraged. The effective dose for a routine CT abdomen-pelvis with contrast is 16 mSV [18] and 11.2 mSV [19] for CTKUB and they pose a significant lifetime risk of cancer [17].

On the other hand, although not significant, appendicectomy does pose potential operative complications. The commonest of which is wound infection and pelvic abscess, the risk of which are 3.3–10.3% and 9.4% respectively [20]. Nonetheless, these complications are minimal in laparoscopic versus open appendicectomies, with a reduced rate of abscess formation which can be as low as 0.002% [21].

Overall, as surgeons we need to reserve the use of appropriate pre-operative imaging for those with an unclear diagnosis and balance its use against the cost and morbidity of laparoscopy alone. It may be that with ever increasing demands on the surgical team that pre-operative imaging may become more prevalent to further reduce the NA rate and reduce the burden in the emergency theatre.

Conversely, negative appendectomies are not necessarily futile. Faecoliths within the appendix may present without radiological evidence of inflammation or appendicitis but can still cause pain and histological diagnosis would show no evidence of appendicitis and therefore be classed as a negative appendicectomy. In these cases, appendicectomy relieves the pain and renders the operation a success [22]. Additionally, appendicectomy in these patients could potentially prevent the inflammatory changes in the appendix due to the obstruction caused by the compacted faecal material [23].

Surgeons should maintain a healthy scepticism of a negative imaging result, particularly following a negative ultrasound. Although the numbers were small false negative results in CTAP, CTKUB and US were 38% (N = 10), 22% (N = 2) and 46% (N = 162) respectively. Additionally, alternative pathology such as neuroendocrine tumors (N = 23) (which may require further excisional surgery), faecoliths (N = 38) and helminth infections (N = 16) could be responsible for further attacks of RIF pain. [quote the rates of each in the study].

We feel that a robust protocol needs to be in place to manage patients with negative scans. They should be given clear instructions to return to the hospital should their symptoms of abdominal pain, fever and vomiting progress, and ideally be reassessed in an acute (hot) surgical follow up clinic within a couple of days. With such follow up in place it would be possible to investigate the role of treating appendicitis by Non-Operative Management with antibiotics alone [24].

Finally, surgeons should not be misled by negative radiological findings of appendicitis when the clinical picture suggests one, as such the diagnosis of appendicitis is still a clinical one and as such patients should be monitored and reviewed if there is clinical doubt and informed to seek medical attention if their symptoms worsen.

## 5 Conclusion

Pre-operative imaging clearly has a role in the diagnosis of appendicitis and in terms of CT has an equivalent NA rate to clinical diagnosis alone. In our centre USS alone has been shown to be a poor tool in the diagnosis of appendicitis and leads to more patients requiring a laparoscopic procedure due to unequivocal results. With an increasing burden of emergency admissions to the secondary care setting appropriate pre-operative imaging would reduce the NA rate and by proxy reduce the strain in the already busy emergency theatre. Moreover, consequences of a surgical intervention to an individual such as financial implications, time off work and therefore economic implications and general recovery and morbidity from an operation and/or complication thereof, need to be borne in mind when deciding upon surgical intervention.

## Provenance and peer review

Not commissioned, externally peer reviewed.

## Ethical approval

No ethical approval sought as anonymised retrospective data.

## Funding

No funding sources.

## Consent

n/a.

## Author contribution

Jaideep Singh Rait – data collection, analysis, interpretation, writing the paper.

Jirayr Ajzajian – data analysis, writing the paper.

Joshua McGillicuddy – data analysis, writing the paper.

Amit Sharma – data collection, data analysis, writing the paper.

Brian Andrews – data analysis, interpretation, editing the paper and supervision.

## Registration of research studies

Research Registry

researchregistry6001

https://www.researchregistry.com/browse-the-registry#home/registrationdetails/5f5912742d4ce10015e697aa/

## Guarantor

Jaideep Singh Rait.

## Declaration of competing interest

No conflicts of interest.
